# Diagnosing Co-Morbid Drug Use in Patients With Alcohol Use Disorders

**Published:** 2008

**Authors:** Bachaar Arnaout, Ismene L. Petrakis

**Keywords:** Alcohol use disorder (AUD), alcohol and other drug disorder (AODD), substance use disorder (SUD), AOD abuse, AOD dependence, substance abuse, substance dependence, co-morbidity, multiple drug use, diagnosis, patient assessment and diagnosis, *Diagnostic and Statistical Manual of Mental Disorders*, *4th Edition* (DSM–IV)

## Abstract

Alcohol and other drug (AOD) use disorders (i.e., AOD abuse and dependence) commonly co-occur. This co-morbidity has important social, psychiatric, and medical consequences. Although making an accurate diagnosis can be challenging, especially in the context of multiple disorders, clinicians can adopt practices to improve their diagnostic accuracy. These practices include an empathic, accepting, and nonjudgmental stance that encourages patients to be honest and forthcoming in their self-report of alcohol use; being sensitive to the prevalence of substance use disorders in all patient populations and settings; and being familiar with diagnostic criteria.

As with alcohol and other drug (AOD) use, AOD use disorders (i.e., abuse and dependence) commonly co-occur and are associated with serious consequences. Making an accurate diagnosis can be complicated, but it is an important first step toward treatment and recovery. After reviewing the prevalence of AOD abuse and dependence co-morbidity and some of the negative social, psychiatric, and medical consequences of co-morbidity, this article presents an overview of how to accurately diagnose a substance use disorder, with a special emphasis on diagnosing drug use disorders in patients who have alcohol use disorders. In addition, it describes some barriers to making a diagnosis and techniques to overcome these barriers. The article focuses on the common drugs of abuse, such as cocaine, opiates, and cannabis. The diagnosis of alcohol use disorders is beyond the scope of this article. For this information, the reader is referred to the National Institute on Alcohol Abuse and Alcoholism’s *Helping Patients Who Drink Too Much: A Clinician’s Guide^1^*. In addition, nicotine use in patients with alcohol use disorders is not addressed because this co-morbidity was the focus of a past issue of *Alcohol Research & Health* (Vol. 29, No. 3, 2006). The recommendations included in this article are oriented toward mental health clinicians but address issues that pertain to most health care settings.

## Prevalence of Co-morbid Substance Use Disorders

**Figure 1:**
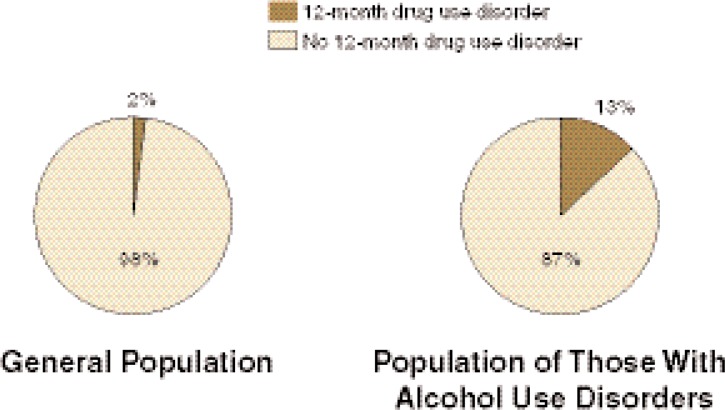
The 12-month prevalence of drug use disorders in the general population (left) and among those with 12-month alcohol use disorders (right). NOTE: 12-Month prevalence represents the prevalence of those meeting the diagnosis for a drug use disorder in the previous 12 months. SOURCE: 2001–2002 National Epidemiologic Survey on Alcohol and Related Conditions (Stinson et al. 2005).

AOD use disorders have a high prevalence in the general population and frequently co-occur. In the 2001–2002 National Epidemiologic Survey on Alcohol and Related Conditions (NESARC), the 12-month prevalence of drug use disorders (i.e., the prevalence of those meeting the diagnosis for a drug use disorder in the previous 12 months) among those with 12-month alcohol use disorders was 13 percent (see figure 1). Conversely, the 12-month prevalence of alcohol use disorders among those with 12-month drug use disorders was 55.17 percent (Stinson et al. 2005). In the general population, the 12-month prevalence of drug use disorders was 2 percent (see figure 1) and the 12-month prevalence of alcohol use disorders was 8.46 percent (Stinson et al. 2005).

**Figure 2:**
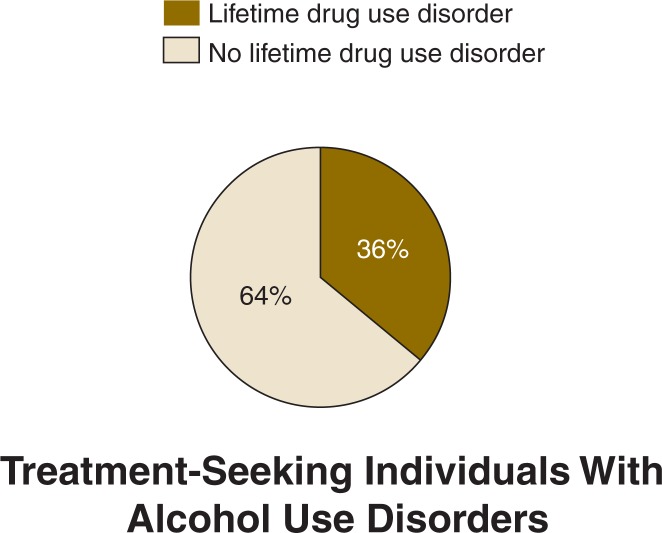
The lifetime prevalence of drug use disorders in a sample of 248 people seeking treatment for an alcohol use disorder (Staines et al. 2001).

Among those with 12-month alcohol use disorders, NESARC reported the following 12-month prevalence rates of specific drug use disorders: sedatives (0.75 percent), tranquilizers (0.85 percent), opioids (2.41 percent), amphetamines (1.22 percent), hallucinogens (1.31 percent), cannabis (9.89 percent), cocaine (2.51 percent), and solvents/inhalants (0.17 percent) (Stinson et al. 2005). Rates of AOD use co-morbidity probably are even higher among patient populations. For instance, in a sample of 248 people seeking treatment for alcohol use disorders, 64 percent had a co-morbid drug use disorder at some point in their lifetime (see figure 2). Sixty-eight percent reported using one or more drugs in the past 90 days, including powder cocaine (33 percent), crack cocaine (29 percent), heroin (15 percent), and cannabis (24 percent) (Staines et al. 2001).

## Consequences of Co-Morbidity

People with both an alcohol use disorder and a co-morbid drug use disorder are more likely to have less education and a lower income and are less likely to be involved in a stable relationship than people who have an alcohol use disorder and no co-morbid drug use disorder (Stinson et al. 2005). In addition, co-morbidity is associated with a higher prevalence of personality, mood, and anxiety disorders (Stinson et al. 2005) and is a predictor for suicide attempts (Borges et al. 2000). AOD use also is associated with a wide array of medical complications (Stein 1999). Not only are individuals at risk for complications from more than one substance (e.g., consequences of cocaine use plus consequences of alcohol use), but AODs used concurrently can interact in complex ways. For example, alcohol may enhance the pleasurable effects of cocaine and result in a larger increase in heart rate than observed with the use of either substance alone (McCance-Katz et al. 1998). In another example, the use of alcohol with other respiratory depressants, such as the benzodiazepines, may result in a synergistic effect and increase the risk of fatal poisoning (Koski et al. 2002). The use of one substance also may worsen the clinical course of the other substance used; for example, in hospitalized patients with alcohol dependence and/or cocaine dependence, postdischarge cannabis use may lead to relapse to alcohol and cocaine use and reduce the likelihood of remission (Aharonovich at al. 2005). Furthermore, alcohol dependence with co-morbid drug dependence is associated with a more severe course than alcohol dependence alone. That is, such patients meet a higher number of criteria from the *Diagnostic and Statistical Manual of Mental Disorders, 4th Edition* (DSM–IV), begin drinking regularly and report being drunk for the first time at an earlier age, and have an early onset of alcohol dependence. These indicators may reflect a more heritable form of alcohol dependence (Dick et al. 2007).

## General Guidelines for Diagnosing a Substance Use Disorder

The information necessary for the diagnosis of a substance use disorder usually is obtained from the patient (i.e., by self-report). Clinicians should ask all patients about past and present substance use. They may further screen for drug problems using validated instruments, such as the Drug Abuse Screening Test (DAST) (Skinner 1982; Staley and El-Guebaly 1990) or the CAGE Adapted to Include Drugs (CAGE-AID^2^) (Brown and Rounds 1995), as well as obtain pertinent biological screening evaluations, such as urine testing. With a positive screen, the clinician can proceed with further history taking. A full history addresses all substances used and includes age at first use; patterns of use, including the amounts and routes of administration; related consequences; periods of abstinence; and the history of treatment and participation in mutual-help recovery. Substance use problems also should be assessed in an orderly sequence. This can be done by either asking the patient to identify the drug that currently is causing him or her the most problems or by assessing the quantity and frequency of each drug being used and establishing a sequential hierarchy to guide the interview. The clinician then can arrive at diagnoses for each category of drug used in a systematic, step-by-step fashion. Evaluating the patient for intoxication and withdrawal is a crucial component of every assessment, as both may require urgent medical or psychiatric care. The diagnosis of AOD use disorders must be made within the context of a larger clinical picture. The full assessment also includes the patient’s psychiatric history, medical history, family history, and social and developmental history. The clinician also should obtain a physical and mental status examination, followed by relevant laboratory and imaging tests. Clinicians may supplement these assessments using structured diagnostic interviews—reliable, albeit time-consuming, instruments for diagnosing AOD use disorders—such as the Structured Clinical Interview for DSM–IV Axis I Disorders, Clinician Version (SCID–CV) (First et al. 1996), the Semi-Structured Assessment for the Genetics of Alcoholism (SSAGA) (Bucholz et al. 1994), and the Semi-Structured Assessment for Drug Dependence and Alcoholism (SSADDA) (Pierucci-Lagha et al. 2005). After obtaining signed patient consent, access to previous records and information from family and friends also can provide valuable information.

## Meeting DSM–IV–TR Criteria

DSM–IV and its text revision (DSM–IV–TR) (American Psychiatric Association 1994 and 2000, respectively) identify substance use disorders as substance dependence or substance abuse and define diagnostic criteria that apply to all substances of abuse.

### Substance Dependence

Substance dependence is defined as a maladaptive pattern of substance use leading to clinically significant impairment or distress, as manifested by the presence of at least three of seven criteria within the same 12-month period. Two of the criteria reflect physiological dependence, defined as tolerance or withdrawal. The remaining five criteria reflect loss of control and adverse consequences:

- Use in larger amounts or over a longer period than was intended;
- A persistent desire or unsuccessful efforts to cut down or control use;
- A great deal of time spent on activities necessary to obtain the substance, use it, or recover from its effects;
- Giving up or reducing important social, occupational, or recreational activities because of use; and
- Continued use despite having a persistent or recurrent physical or psychological problem that is likely to have been caused or exacerbated by the substance.

In addition, DSM–IV–TR identifies six criteria for substance dependence that are meant to aid in the identification of different subgroups of patients. Four of these “course specifiers” define types of disorder remission (i.e., early full remission, early partial remission, sustained full remission, and sustained partial remission), and two additional specifiers are applied if the patient is receiving a prescribed medication that mimics the substance of abuse (i.e., agonist therapy) or is currently living in a controlled treatment environment.

### Substance Abuse

Substance abuse is defined as a maladaptive pattern of substance use leading to clinically significant impairment or distress, as manifested by the presence of at least one of four of the following criteria within a 12-month period:

- Failure to fulfill major role obligations;
- Recurrent use in situations in which it is physically hazardous;
- Recurrent substance-related legal problems; and
- Continued use despite having persistent or recurrent social or interpersonal problems caused or exacerbated by the effects of the substance.

If criteria for both substance abuse and substance dependence are met, the diagnosis of substance dependence should be given. If criteria for more than one substance use disorder are met, all individual diagnoses should be documented (e.g., alcohol dependence, cocaine dependence, heroin dependence, and phencyclidine abuse). However, if a patient repeatedly uses at least three groups of substances (not including caffeine and nicotine), with no single substance predominating, and the criteria for substance dependence are met for these substances as a group but not for any specific substance, the patient is diagnosed with polysubstance dependence. The commonly used term polysubstance abuse is not a valid DSM–IV–TR diagnosis.

## Barriers to Accurate Diagnosis of Substance Use Disorders

Although accurate identification facilitates timely intervention, substance use disorders often go undetected (Stein et al. 1996), and establishing an accurate diagnosis can be challenging. Issues that can complicate an accurate diagnosis include (1) patient misreport, (2) clinician misrecognition, and (3) challenges related to use of the DSM–IV–TR. Naturally, this distinction is arbitrary, and there is considerable overlap between these three areas. It is our intention here, however, to describe the interplay between the three components of the diagnostic interaction: the patient, the clinician, and the diagnostic system used.

### Patient Misreport

Information gathered from patients regarding substance use usually is accurate. Nevertheless, patients may underreport or conceal their substance use. Generally, patients are more willing to discuss their use of legal drugs such as alcohol and nicotine than their use of illicit drugs. As demonstrated by Harrell (1997), underreporting of drug use correlates with the social stigma associated with the drug. Patients are less likely to accurately report use of heroin, followed by cocaine and hallucinogens; they are relatively more likely to report use of cannabis. In practice, many patients engaged in substance abuse treatment confirm that their underreporting of substance use to health care professionals often is driven by fear of receiving poor care if labeled as “addicts” or “junkies.” Patients also may fear that their substance use will be revealed to their families and significant others or may lead to legal consequences. Despite these concerns, the validity of self-reported drug use in patients with alcohol use disorders has been shown to be relatively high (Staines et al. 2001).

Fortunately, there are steps clinicians can take to increase the accuracy of self-reported substance use. Before beginning the interview, the clinician should be familiar with Federal and State regulations regarding confidentiality. Informing the patient of the scope of confidentiality and ensuring privacy also is likely to facilitate open communication. Using an empathic, accepting, and nonjudgmental approach during the interview also can help reduce the patient’s fear of stigmatization and discrimination and facilitate valid self-report. Asking questions in a direct manner communicates that the clinician is comfortable with assessing AOD use. How the question is framed also is important. Asking a patient, “You don’t use drugs, do you?” implies that the “right” answer is “no.” A question more likely to elicit an accurate response is, “Tell me about your drug use.” It also is useful to inquire about the use of individual substances; patients may not regard cannabis and prescribed medications as “drugs.”

Denial and resistance can therefore be viewed as part of the interpersonal dynamic between patient and clinician. That is, the patient’s resistance to discussing his or her AOD use often is a mirror image of the clinician’s discomfort in dealing with the issue of substance abuse. By becoming more familiar with motivational interviewing (MI) (Miller and Rollnick 2002) clinicians can overcome this common barrier. MI is an evidence-based therapeutic approach. It offers a method for interacting with people that facilitates open communication. In MI, the interview takes place in a spirit of collaboration, with emphasis on evoking the patient’s resources and motivation for change, while maintaining his or her autonomy. The clinician asks open-ended questions, listens reflectively, provides the patient with affirmation and support, and periodically summarizes gathered information. Eventually, a plan of action is developed. Without the pressure to convince or “fix” the other person, a space is created where substance use can be talked about openly, creating a relaxed environment for both patient and clinician. This style not only is likely to facilitate communication but also helps patients resolve any ambivalence they may have and enhance their motivation to change. The use of MI has been expanding. MI-based strategies have been adapted to provide brief intervention in emergency department settings (Academic ED SBIRT Research Collaborative 2007), as well as to assess and provide brief intervention to patients in primary-care settings (Botelho and Novak 1993).

Although less common, patients sometimes may overreport their substance use. For example, a patient may overreport opioid use and related consequences to gain access to agonist treatment with methadone or buprenorphine. As suggested above, the clinician should retain a nonjudgmental stance and thoroughly assess the patient’s situation and the reason for his or her overreporting.

To ensure accuracy, the clinician should seek to supplement the patient’s reports of substance use with information from medical records and family members or friends. A patient who is experiencing inadequately treated pain may overreport substance use and may need a referral to a pain specialist rather than agonist treatment in a substance abuse treatment setting. In such cases, the clinician still can provide the patient with adequate treatment or referral, and this can be done in a way that is not embarrassing to the patient.

#### Special Considerations in Patients With Alcohol Use Disorders

Patients who have been diagnosed with alcohol use disorders, and who may be enrolled in substance abuse treatment, also may misreport their substance use, usually for the same reasons as outlined above. Even patients who identify alcohol as a problem may feel that use of substances that are less likely to cause a withdrawal syndrome, such as marijuana, is acceptable. Further, substances used intermittently, such as weekend cocaine use, also may be ignored or underreported. Use of benzodiazepines or oral opioids may be viewed as therapeutic even if not prescribed, and patients may not view their use as drug abuse. Patients who already are enrolled in treatment for alcohol use disorders also may begin using other substances, even while abstaining from alcohol. These patients may either not identify the use as problematic, as described above, or may be embarrassed about their use and therefore conceal this information from the clinician. The clinician can elicit accurate information by asking if the patient has ever used any other substances and then follow up by specifically asking about each major category of abused substances.

For example, a woman in her forties, interviewed by one of the authors (B.A.) at a residential treatment program, gave a history of alcohol and cocaine dependence and denied using any other substances. When asked specifically for major classes of abused substances, she reported that she sometimes uses marijuana. When asked to elaborate, she responded that she uses marijuana “once in a blue moon.” When asked again how often that is, she reported smoking marijuana 1 to 2 days per week. She also gave a history of heavy cigarette smoking.

In this example, the patient was forthcoming in discussing her alcohol and cocaine use but did not initially view her use of marijuana and nicotine as important to mention or address. As noted earlier, marijuana use may lead to relapse to alcohol and cocaine use and reduce the likelihood of remission (Aharonovich at al. 2005).

### Clinician Misrecognition

As described in the previous section, overcoming both patients’ and clinicians’ resistance is very important. To overcome the “wall of silence” associated with substance use disorders in general, and those related to illicit substances in particular, clinicians should openly evaluate substance use in all patients, regardless of patient characteristics and treatment settings. Clinicians may be less likely to ask about substance use when assessing patients who do not conform to common perceptions of a typical substance user. For example, because of the generally higher prevalence of substance use disorders in men, clinicians may under-recognize substance use disorders in women. This may be further complicated by the social stigma attached to substance use in women (Blume 1990). At the same time, gender differences in rates of substance use disorders gradually are diminishing, especially among younger people (Greenfield 2002). In addition, substance-dependent women appear to experience a more rapid progression of the disorder (i.e., “telescoping”) (Hernandez-Avila et al. 2004), which further emphasizes the need for early and accurate diagnosis.

In reality, there is no typical substance abuser. Data from NESARC demonstrate that AOD use disorders affect people across genders, age-groups, and ethnic backgrounds (Stinson et al. 2005). Although there are differences in the prevalence of substance use disorders related to gender, age, and ethnicity, these prevalence rates often change over time. Further, demographic variables are of questionable value when used to assess any one patient.

Although demographic variables can be misleading when diagnosing substance use disorders, knowledge of drug availability may help the clinician when taking the patient’s history. The prevalence of specific AOD use disorders varies by geographic location. Alcohol is the most prevalent psychoactive substance used (other than caffeine and nicotine), whereas use of other drugs varies by region and availability. For example, cocaine use is especially problematic in eastern parts of the United States and the Great Lakes region, whereas methamphetamine use is higher in the western and central States and is becoming increasingly problematic in the eastern States (National Drug Intelligence Center 2006). Use of heroin is especially prevalent in large metropolitan areas; however, the widespread use of prescription pain medications has made illegitimate opioid use a problem in previously less affected areas (National Drug Intelligence Center 2007). Finally, some patient populations may have high rates of disorders that do not reflect the patient population at large. For example, in methadone maintenance treatment (MMT), cocaine dependence may be more frequent than alcohol dependence (Brooner at al. 1997). As mentioned above, this information might help guide clinicians, but such stereotypes of drug patterns should not limit a full evaluation for any individual patient.

Settings also should be considered when assessing AOD use. Research shows that clinicians are less likely to recognize substance use disorders in nonsubstance abuse treatment settings such as mental health treatment settings (Carey and Correia 1998) or primary-care settings (Coulehan et al. 1987; Duszynski et al. 1995). In these settings, patients often are seeking help for other disorders and may not identify substance use as a problem. Patients may resent questions about AOD problems, feeling that this detracts from the problem at hand. Further, clinicians in those settings often have little formal training in diagnosing or assessing substance use disorders and may feel uncomfortable asking questions about AOD use. In reality, clinicians often encounter patients with substance use disorders, as the prevalence in the treatment-seeking population exceeds community estimates (Stein et al. 1996). Therefore, the presence of one disorder, whether medical or psychiatric, should prompt the clinician to assess every patient for a substance use disorder.

#### Special Considerations in Patients With Alcohol Use Disorders

As described above, and as recognized by many clinicians, patients with an alcohol use disorder also are likely to have a coexisting drug use disorder. However, clinicians may have a bias against asking patients about drug use, particularly illicit drug use. The clinician may not even consider asking about other drug use if a patient already has been identified with an alcohol use disorder. This may be particularly evident in patients already engaged in treatment for alcohol use disorders, for whom drug use was not previously identified. In addition, patients in treatment for alcohol use disorders may not be asked to undergo routine urine drug screening because this laboratory test does not screen for alcohol use. Hence, early detection may be missed. Similarly, clinicians, particularly nonphysicians, may be reluctant to ask about prescription medications and therefore miss benzodiazepine or oral opioid use. In assessing patients with alcohol use disorders, clinicians should take full drug histories, including use of prescription drugs. In addition, urine drug tests should be routine. In patients already enrolled in treatment, it is reasonable to conduct periodic reassessment of AOD use. This could include urine monitoring for drug use. Making this explicit at the start of treatment and stressing that such tests are an important component of successful substance abuse treatment will help establish a therapeutic “contract” and establish the need for routine testing even if the patient reports doing well in treatment.

### Challenges Related to DSM–IV–TR

Diagnosing substance use disorders based on the DSM–IV–TR criteria poses challenges that may hamper accurate diagnosis or assessment. For example, deciding between a diagnosis of substance abuse or substance dependence can be difficult, as some of the criteria overlap. Another challenge lies in distinguishing between physiological dependence (i.e., tolerance and withdrawal) and the DSM–IV–TR diagnosis of substance dependence. Because the DSM–IV–TR requires the presence of three out of seven criteria, physiological dependence alone is not sufficient for a diagnosis of substance dependence. For example, a patient on long-term opioid therapy for chronic pain will have developed physiological dependence but does not meet criteria for the DSM–IV–TR diagnosis of substance dependence unless at least one additional criterion of substance dependence is present. Conversely, physiological dependence is not necessary for a DSM–IV–TR diagnosis of substance dependence. As an example, the use of hallucinogens or binge drinking of alcohol may not lead to tolerance or withdrawal, but the presence of any other three out of seven criteria establishes the diagnosis of substance dependence. Nevertheless, the presence of tolerance and withdrawal should alert the clinician that the patient may have a DSM–IV–TR substance dependence diagnosis, in which case the disorder is associated with greater severity (Schuckit et al. 1999).

Additional challenges lie in assessing patients whose substance use is hazardous or harmful but does not meet the criteria for a DSM–IV–TR diagnosis of substance abuse or dependence. For example, a patient who has used cocaine several times might not have developed clinically significant impairment or distress to qualify for a DSM–IV–TR diagnosis. This should not prevent the clinician from thoroughly evaluating the patient, providing preventive measures and early intervention, and following the course of use over time. The clinician should be especially concerned when a patient reports beginning substance use at an early age, which is a predictor of developing a substance use disorder as well as a predictor of higher disorder severity (Robins and Przybeck 1985). Further, substance use disorders change over time, so clinicians should remain flexible and make diagnostic revisions when necessary.

Classification systems other than the DSM–IV–TR also may be used in diagnosing substance use disorders. For example, the *10th Revision of the International Statistical Classification of Diseases and Related Health Problems* (ICD–10) (World Health Organization 1992) is widely used in many countries and often corresponds to the DSM–IV–TR. Other medical specialties may use different language and criteria to define and diagnose substance use disorders. At the same time, knowledge of DSM–IV–TR criteria is important, as these indicators continue to be the standard in diagnosing substance use disorders in the United States, especially by mental health professionals.

#### Special Considerations in Patients With Alcohol Use Disorders

Patients with alcohol use disorders and other co-occurring substance use may experience adverse consequences in a wide range of areas, affecting social, mental, and physical functions. It can be difficult to determine whether that loss of function is related to all or some of the substances being used. For example, a patient may attribute his or her problems to the continued use of alcohol and heroin but may deny or not recognize the adverse consequences related to his or her biweekly cocaine use. The clinician certainly should address the patient’s use of cocaine, but deciding whether it technically qualifies for a DSM–IV–TR diagnosis and, if so, whether it is cocaine abuse or dependence may not be a central issue. Instead, clinicians should document diagnostic dilemmas and use good clinical judgment to address *all* used substances, while at the same time following the course of illness over time, making diagnostic revisions, and providing adequate treatment or referral.

## Summary

Co-morbidity of drug use in patients with alcohol use disorders is common and has important social, psychiatric, and medical consequences. Accurately diagnosing substance use disorders can be complicated, especially when related to substances associated with a social stigma. This is true even for patients in whom an alcohol use disorder has been identified. Several factors may help clinicians to improve diagnostic accuracy: adopting an empathic, accepting, and nonjudgmental stance to encourage patients to be accurate and forthcoming in their reports of AOD use; being sensitive to the prevalence of substance use disorders in all patient populations and settings; and being alert to nuances of DSM–IV–TR diagnostic criteria. A full assessment provides a wealth of findings that should be considered within the context of the patient’s full clinical picture. In addition, clinicians should remain flexible when following the course of illness over time. Adhering to these common practices will help the clinician to make an accurate first diagnosis and to revise that diagnosis when necessary over the course of the illness, providing the best prospects for successful treatment and referral.

## Footnotes

1 More information on *Helping Patients Who Drink Too Much: A Clinician’s Guide* can be found at www.niaaa.nih.gov/guide.

2 The CAGE is a four-question instrument with each question representing a letter in the acronym. The CAGE questions Adapted to Include Drugs (CAGE-AID) are: Have you felt you ought to **C**ut down on your drinking or drug use? Have people **A**nnoyed you by criticizing your drinking or drug use? Have you felt bad or **G**uilty about your drinking or drug use? Have you ever had a drink or used drugs first thing in the morning to steady your nerves or to get rid of a hangover (**E**ye-opener)?
